# Comparison of Clinically Relevant Oncolytic Virus Platforms for Enhancing T Cell Therapy of Solid Tumors

**DOI:** 10.1016/j.omto.2020.03.003

**Published:** 2020-03-19

**Authors:** Victor Cervera-Carrascon, Dafne C.A. Quixabeira, Riikka Havunen, Joao M. Santos, Emma Kutvonen, James H.A. Clubb, Mikko Siurala, Camilla Heiniö, Sadia Zafar, Teija Koivula, Dave Lumen, Marjo Vaha, Arturo Garcia-Horsman, Anu J. Airaksinen, Suvi Sorsa, Marjukka Anttila, Veijo Hukkanen, Anna Kanerva, Akseli Hemminki

**Affiliations:** 1 Cancer Gene Therapy Group, Translational Immunology Research Program, University of Helsinki, 00290 Helsinki, Finland; 2 TILT Biotherapeutics, 00290 Helsinki, Finland; 3 Department of Chemistry, Radiochemistry, University of Helsinki, 00560 Helsinki, Finland; 4 Regenerative Pharmacology Group, Division of Pharmacology and Pharmacotherapy, University of Helsinki, 00560 Helsinki, Finland; 5 Pathology, Finnish Food Authority, 00790 Helsinki, Finland; 6 Institute of Biomedicine, University of Turku, 20500 Turku, Finland; 7 Department of Obstetrics and Gynecology, Helsinki University Central Hospital, 00290 Helsinki, Finland; 8 Helsinki University Hospital Comprehensive Cancer Center, 00290 Helsinki, Finland

**Keywords:** oncolytic virus, immunotherapy, T cell therapy, tumor microenvironment, solid tumor, gene therapy, adenovirus, vaccinia virus, herpes simplex virus, reovirus

## Abstract

Despite some promising results, the majority of patients do not benefit from T cell therapies, as tumors prevent T cells from entering the tumor, shut down their activity, or downregulate key antigens. Due to their nature and mechanism of action, oncolytic viruses have features that can help overcome many of the barriers currently facing T cell therapies of solid tumors. This study aims to understand how four different oncolytic viruses (adenovirus, vaccinia virus, herpes simplex virus, and reovirus) perform in that task. For that purpose, an immunocompetent *in vivo* tumor model featuring adoptive tumor-infiltrating lymphocyte (TIL) therapy was used. Tumor growth control (p < 0.001) and survival analyses suggest that adenovirus was most effective in enabling T cell therapy. The complete response rate was 62% for TILs + adenovirus versus 17.5% for TILs + PBS. Of note, TIL biodistribution did not explain efficacy differences between viruses. Instead, immunostimulatory shifts in the tumor microenvironment mirrored efficacy results. Overall, the use of oncolytic viruses can improve the utility of T cell therapies, and additional virus engineering by arming with transgenes can provide further antitumor effects. This phenomenon was seen when an unarmed oncolytic adenovirus was compared to Ad5/3-E2F-d24-hTNFa-IRES-hIL2 (TILT-123). A clinical trial is ongoing, where patients receiving TIL treatment also receive TILT-123 (ClinicalTrials.gov: NCT04217473).

## Graphical Abstract

**Figure:**
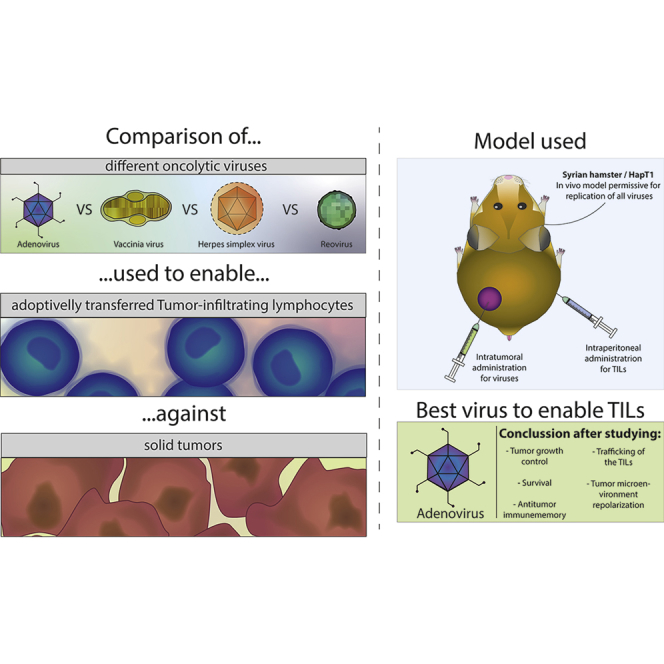

## Introduction

The fundamental idea of viral infections being beneficial for tumor control has been present for over 100 years,^1^ originating just a few years after viruses were discovered. Already on a theoretical level, it makes sense to use those agents to treat cancer, as the cellular and molecular characteristics of tumor cells make them particularly vulnerable to viral infections. Fast growth and defects in apoptosis and immune mechanisms are some of the key flawed mechanisms in cancer cells, making it more difficult for them to stop virus infections.^2^ In 1949,^3^ the first one using a virus as an antitumor therapy was carried out, and in the following years, other studies using viruses for the treatment of different tumor types were conducted.^4^, ^5^, ^6^ However, the overall inconclusive results, influenced by small patient numbers and poor “products” (mainly consisting of wild-type viruses), reduced the interest of the scientific community in virotherapy of cancer. Later, technological development allowed the refinement of the approach by the engineering of tumor-selective viruses.^7^

Because the efficacy of single-agent virotherapy has generally been suboptimal, immunosuppressive chemotherapy has been studied preclinically for its ability to increase direct oncolytic activity.^8^, ^9^, ^10^, ^11^, ^12^ When it was realized that in humans, oncolytic viruses trigger anti-tumor immunity, the approach was radically changed.^13^ Instead of aiming at optimal oncolysis, viruses were now being designed to enhance immune activities. The first oncolytic virus approved in the United States and Europe,^14^ talimogene laherparepvec (Imlygic), is a herpes simplex virus (HSV) engineered to express granulocyte-macrophage colony-stimulating factor (GM-CSF). talimogene laherparepvec, other oncolytic viruses have also been designed to include immunostimulatory gene constructs, such as cytokines, ligands, or antagonists.^15^, ^16^, ^17^

In the last decade, the T cell component of the immune system has been drawing attention, as T cell-related therapies, such as chimeric antigen receptor T cells (CAR Ts),^18^^,^^19^ adoptive T cell therapy with tumor-infiltrating lymphocytes (TILs),^20^^,^^21^ adoptive T cell receptor therapy,^22^ and checkpoint inhibitors^23^ emerged as potent tools in clinical oncology. These approaches have been used in the clinics with promising results, as they can result in long-lasting responses and in fact, appear to be able to cure a proportion of patients with metastatic cancer. Consequently, several products have been approved.

On the other hand and especially regarding solid tumors, only a fraction of treated patients benefit from these therapies and only in particular indications. Overall response rates are below 10% for CAR T therapy,^24^ around 50% for adoptive T cell therapy of melanoma,^25^ and 0%–40% for checkpoint inhibitors.^26^ Most commonly, T cell therapies fail because of immunosuppressive conditions in the tumor milieu,^27^^,^^28^ the ability of some tumors to prevent T cell infiltration,^29^^,^^30^ and antigen loss for those treatments where specific targets were chosen for engineered T cells.^31^

Due to their mechanism of action, oncolytic viruses have been proposed as a valuable tool to overcome T cell therapy limitations^15^^,^^32^ and actually have been tested by different groups.^33^^,^^34^ Their ability to create an immunostimulatory signal increases immune cell trafficking toward the tumor,^35^^,^^36^ reverses the immunosuppressive status of the microenvironment,^37^^,^^38^ and creates *de novo* adaptive immunity against the pool of tumor epitopes released upon oncolysis.^39^, ^40^, ^41^ By taking into account that different viruses have different properties, each virus will probably offer distinct therapeutic possibilities. In this study, four different viruses representing different families (*Adenoviridae*, *Poxviridae*, *Herpesviridae*, and *Reoviridae*), widely studied clinically, were chosen for study in the context of adoptive T cell therapy.

## Results

### Selecting Treatment Dose for Different Viruses

The Syrian hamster model was selected to study *in vivo* efficacy, as it is perhaps the only model permissive for the productive replication of all viruses used in the study.^42^, ^43^, ^44^, ^45^ The selected tumor model, HapT1 pancreatic carcinoma, enables the isolation of TILs for *ex vivo* amplification for use as an adoptive cell therapy (ACT).^46^ It was also assessed that the selected virus had oncolytic activity on the cell line (Figure S1).

For the comparison of adenovirus, vaccinia virus, herpes simplex virus, and reovirus, weight-per-weight hamster doses were established based on the maximum tolerated dose used in human trials. At the beginning of the project (May 2016), a search for clinical trials investigating the above-mentioned viruses was performed, and the maximum tolerated dose in humans was identified for each of the viruses (Table 1). Because there was no established maximum tolerated dose for an unarmed herpes simplex virus, that of talimogene laherparepvec was selected for this study.^47^

**Table 1:** Viral Dose Extrapolation According to Maximum-Tolerated Doses in Humans

Virus	Reference	Year	Maximum Dose (Human)	Extrapolated Dose (Hamster)
Adenovirus	Small et al.^71^	2006	6 × 10^12^ vp	8 × 10^9^ vp
Vaccinia	Zeh et al.^72^	2015	3 × 10^9^ PFU	4 × 10^6^ PFU
Herpes simplex	Andtbacka et al.^47^	2015	4 × 10^8^ PFU	5.3 × 10^5^ PFU
Reovirus	Karapanagiotou et al.^73^	2012	3 × 10^10^ TCID_50_	4 × 10^7^ TCID_50_

vp, viral particle; PFU, plaque-forming unit; TCID_50_, median tissue-culture infectious dose.

The dose extrapolation from the human maximum-tolerated dose to the one used in Syrian hamsters assumes 75 kg as standard human weight and 0.1 kg as standard Syrian hamster weight. Thus, maximum tolerated doses in humans were divided 750 times to achieve the dose to be used in hamsters. To assess the feasibility of the extrapolated doses, ten times higher dose and ten times smaller dose were also tested *in vivo* (Figure S2). None of the groups treated with 10 times more virus showed better tumor growth control than the directly extrapolated dose (presumably because of virus replication decreasing the importance of input dose), supporting the rationale for using the extrapolated doses. The use of the same units (viral particle [vp], plaque-forming unit [PFU], or median tissue-culture infectious dose [TCID_50_]) as had been published in human trials avoided the problem of different titering procedures.

### Oncolytic Adenovirus Has the Best Antitumor Efficacy When Used as a T Cell Therapy Enabler

**Figure 1:**
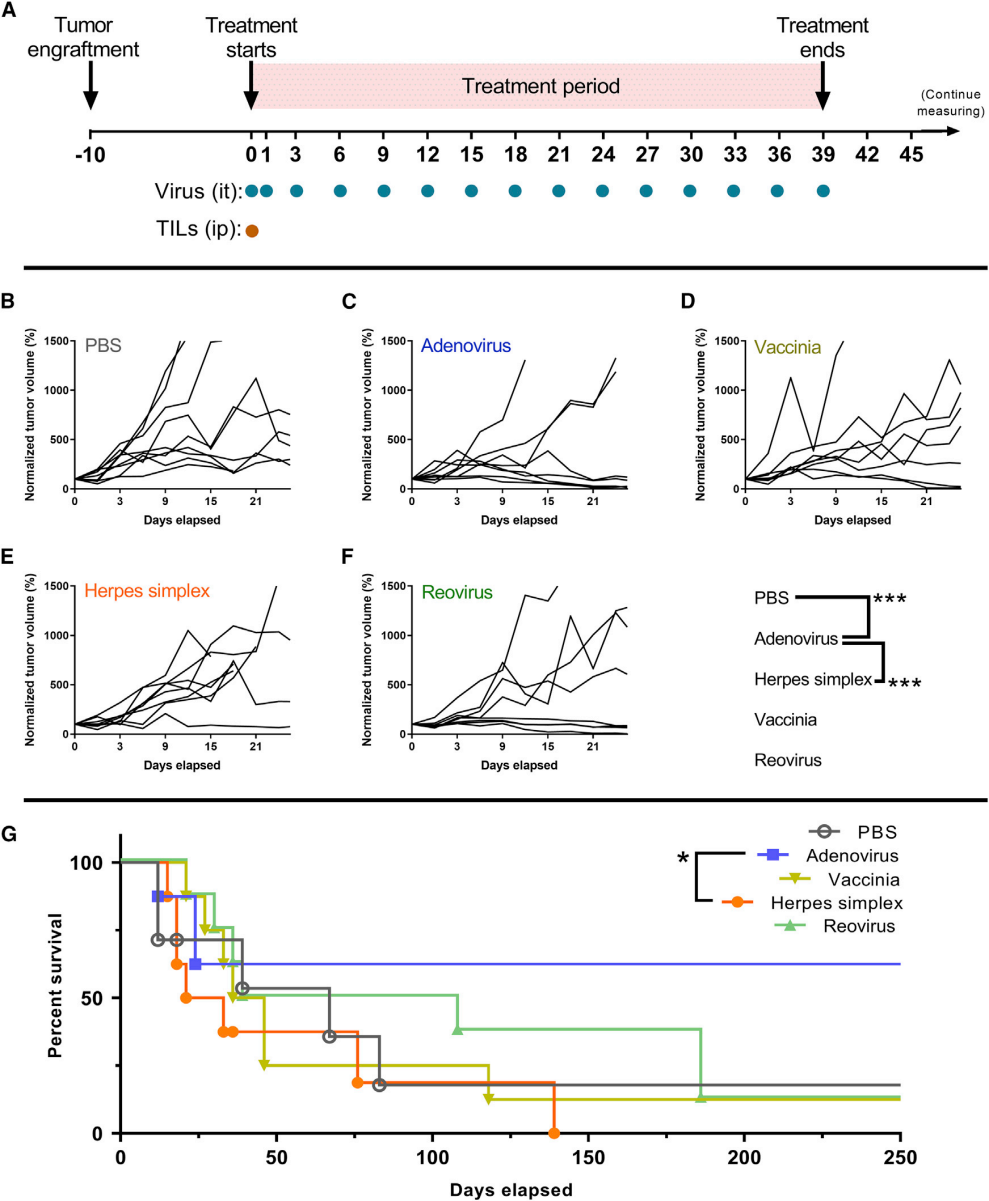
Antitumor Efficacy after the Use of Different Oncolytic Viruses to Enable T Cell Therapy (A) Experimental design: forty-one Syrian hamsters carrying subcutaneous HapT1 tumors were randomized into groups and treated with TILs intraperitoneally and PBS or one of the studied viruses intratumorally. After the treatment period, the animals were followed up to day 250. (B) Individual normalized tumor volume lines for PBS group (n = 9). (C) Individual normalized tumor volume lines for Adenovirus group (n = 8). (D) Individual normalized tumor volume lines for Vaccinia group (n = 8). (E) Individual normalized tumor volume lines for Herpes simplex group (n = 8). (F) Individual normalized tumor volume lines for Reovirus group (n = 8).

For the study of the antitumor efficacy of different viruses, HapT1 cells were subcutaneously engrafted in the lower right flank of Syrian hamsters. Ten days later, when tumors were palpable and measurable (mean volume: 205.63 mm^3^, standard error of the mean: 15.76 mm^3^), those animals were randomized into groups. All animals received an adoptive cell graft of *ex vivo*-expanded TILs intraperitoneally at day 0. Depending on the group, the animals also received intratumoral virus treatments or PBS as a negative control. Intratumoral treatments were administered on days 0, 1, and 3 and then once every 3 days until day 39 (Figure 1A). After day 39, animals were not treated, but regular measuring and health checkups were performed. Animals were kept alive until their tumor exceeded the allowed tumor dimensions (22 mm for the longest tumor diameter).

Individual values of normalized tumor volumes are displayed in Figures 1B–1F. The only virus that provided a significant reduction in tumor volume compared to the effect of adoptive T cell therapy alone was adenovirus (p < 0.001). The group treated with adenovirus also showed significantly better tumor growth control than those animals treated with herpes simplex virus (p < 0.001). None of the other viruses were able to provide significant tumor growth control compared to the PBS control or other viruses.

In line with tumor growth control results, the only group that showed significantly increased survival was the one treated with adenovirus (Figure 1G), when compared with the herpes simplex virus group (p = 0.049). It is also relevant to look at the proportion of complete responses by day 250: PBS (17.5%), adenovirus (62.5%), vaccinia virus (12.5%), herpes simplex virus (0%), and reovirus (12.5%). There were three times more complete responses in the adenovirus group than in the second-best group.

### Antitumor Memory Can Be Elicited by the Use of Oncolytic Adenovirus and T Cell Therapy

**Figure 2:**
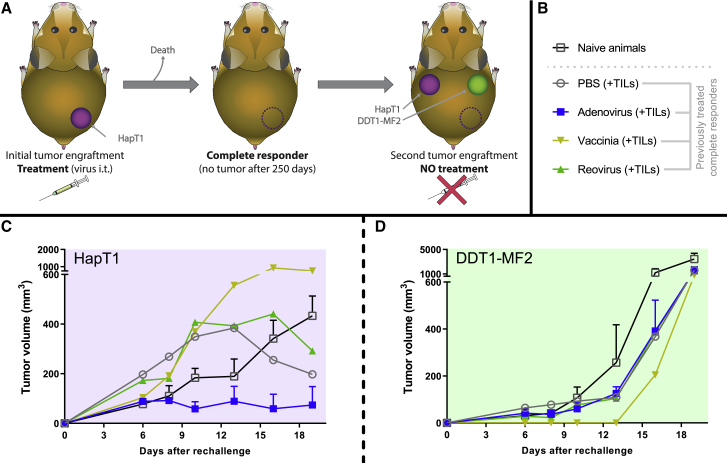
Study of Antitumor Memory in Complete Responders (A) Experimental design: animals treated with ACT and different oncolytic viruses (or PBS) experiencing complete responses were rechallenged with HapT1 and challenged with DDT1-MF2 to study antitumor-specific memory (no additional treatments given). In addition, naive animals were simultaneously engrafted with HapT1 and DDT1-MF2 tumors. (B) Groups included in the experiment: naive animals group (n = 3), PBS + TILs group (n = 1), adenovirus + TILs group (n = 5), vaccinia + TILs (n = 1), and reovirus + TILs (n = 1). The number of animals depended on how many had been cured in the first part of the experiment. (C) Mean (and SEM) tumor volumes for the HapT1 tumors. (D) Mean (and SEM) tumor volumes for the DDT1-MF2 tumors.

After initial treatment, animals that showed no visible tumors by day 250 were included in a follow-up experiment to investigate if they had gained specific antitumor memory able to reject a new graft of the same HapT1 tumors from which they were cured. For that purpose, the same amount of HapT1 cells was engrafted in the opposite side of the hamster’s back (upper-left flank). At the same time, a different cell line (DDT1-MF2, Syrian hamster leiomyosarcoma), to which the animals were naive, was engrafted in the upper-right flank to control the specificity of the antitumor memory. After this tumor rechallenge, the animals did not receive any treatment, as the purpose was to study the ability of the previous treatments to generate immunological memory. Hamsters that were never exposed to any cancer cell lines and/or treatments served as negative control (Figures 2A and 2B). After tumor engraftment, the animals were followed for 19 days. By that day, DDT1-MF2 tumors were reaching the regulatory tumor-size limit in the majority of the animals.

As displayed in Figure 2C, all animals (with the exception of the animal from the vaccinia virus group) that showed a complete response from the previous treatments had lower tumor volumes on day 19 than naive animals. The animals that had previously received adoptive T cell therapy and either PBS or reovirus started developing the tumors faster in the first days of the experiment but then showed noticeable partial responses by day 19. A much clearer result was observed from the animals previously treated with adoptive T cell therapy and adenovirus, as those animals showed lower average tumor volume than naive animals as early as by day 8. By day 19, 5 out of 6 animals in that group showed no visible HapT1 tumor. This result suggests that the adenovirus was most effective in generating antitumor memory. The animal cured with vaccinia virus and T cell therapy had a larger tumor than naive animals, but drawing conclusions on just one animal is difficult. There were no animals cured with Herpes, and thus rechallenge could not be performed.

DDT1-MF2 tumors took a longer time to start growing visibly, but then they increased their volume exponentially (Figure 2D). None of the animals included in the experiment showed protection against this cell line, validating the assumption that rechallenged complete responders had antitumor memory against HapT1 specifically.

### Antitumor Efficacy Is Not Directly Explained by Trafficking of the T Cell Graft

**Figure 3:**
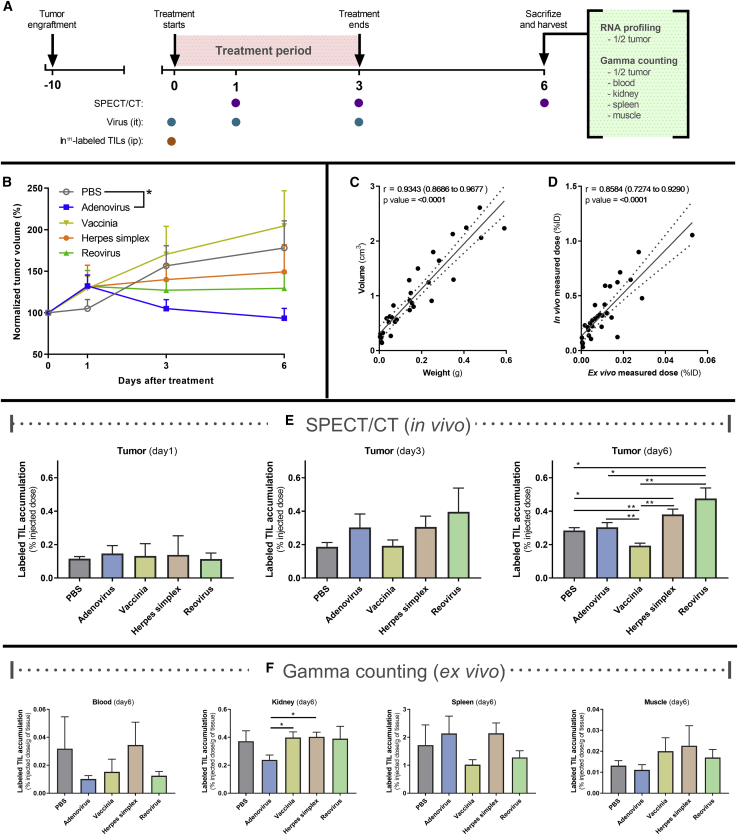
Tracking of Systemically Administered TILs after Oncolytic Virus Intratumoral Injection (A) Experimental design: thirty-four Syrian hamsters carrying subcutaneous HapT1 tumors were randomized into groups and treated with ^111^indium-labeled TILs intraperitoneally and PBS or one of the studied viruses intratumorally. During the experiment, animals were imaged with SPECT/CT to quantify the biodistribution of the injected TILs. At the end of the experiment, animals were euthanized, and different organs were harvested for *ex vivo*^111^indium measurement or in the case of tumors, also for multiplexed RNA sequencing. (B) Normalized tumor volumes for the different groups (n = 6–8). (C) Correlation between tumor volumes measured using CT and the weight of those tumors after they were harvested. (D) Correlation between the *in vivo* radiation signal measured with SPEC/CT and the *ex vivo* samples measured by gamma counting. (E) TIL-associated radiation of tumors measured *in vivo* with SPECT/CT on days 1, 3, and 6. (F) TIL-associated radiation measured *ex vivo* on different tissues by gamma counting on day 6 (∗p < 0.05; ∗∗p < 0.01). All error bars are SEM; ∗p < 0.05; ∗∗p < 0.01.

Our next goal was to study the contribution of the transferred T cell graft to the response and repolarization of the immune microenvironment. For that purpose, a new animal experiment was set up, following similar conditions as before. The main differences were the length of the study and the primary endpoint: tumors were collected 6 days after treatment start to study the biodistribution of the adoptive T cells and gene-expression profiles (Figure 3A).

To study the biodistribution of the T cell graft, the cells were labeled with ^111^indium (^111^In)-oxine. ^111^Indium-oxine is a radioactive compound that allows the tracking of cells *in vivo* with single-photon emission computed tomography (SPECT)/computed tomography (CT) measurements, as well as *ex vivo* radioactivity measurements after organs are collected. At days 0, 1, 3, and 6, tumors were measured with a digital caliper (Figure 3B). In addition, tumor volume was determined on day 6 based on CT images and validated by correlating with the tumor mass after harvesting (Figure 3C). The *in vivo* radioactivity measurements observed with SPECT/CT were also validated by comparing the values to the radiation measured by a gamma counter after tissue harvesting (Figure 3D). A clear correlation between *in vivo* and *ex vivo* radiation uptake was observed (p < 0.0001). Thus, SPECT/CT measurements can be used reliably when evaluating the trafficking of adoptively transferred T cells in different groups.

*In vivo* measurements of the animals were performed approximately 24 (day 1), 72 (day 3), and 144 (day 6) h after the labeled cells were transferred to the animals. A graphic representation of the amount of radioactive signal is shown in Figure 3E. For days 1 and 3, there was no statistically significant increase in the radioactivity levels, but at day 3, a trend indicating reovirus recruiting a higher number of radiolabeled T cells in tumors was observed. At day 6, a significant increase of radioactivity signals was found in tumors treated with reovirus (p = 0.028) and with herpes simplex virus (p = 0.031) when compared with PBS. On the other hand, the vaccinia virus-treated group had lower radioactivity signals than any other group (p < 0.01). The reovirus-treated group also showed significantly higher radioactive-intensity signals than the adenovirus-treated one (p = 0.042). However, the groups having the highest T cell trafficking did not match the groups having the best antitumor response or immunological memory against the tumors.

Besides tumors, other tissues, including blood, kidney, spleen, and muscle, were analyzed *ex vivo* to have a deeper understanding of the biodistribution of the T cell graft. In addition, we wanted to assess if any of the viruses caused extratumoral accumulation of adoptively transferred T cells (Figure 3F). Kidneys in the adenovirus group had lower radioactivity than all of the other groups, and the difference was statistically significant when compared with vaccinia and herpes simplex virus groups (p < 0.05). Also, a considerable proportion of the T cell graft could be found in spleens for all of the groups regardless of the treatments.

### Immune Repolarization Is Seen in the Groups with the Best Antitumor Responses

**Figure 4:**
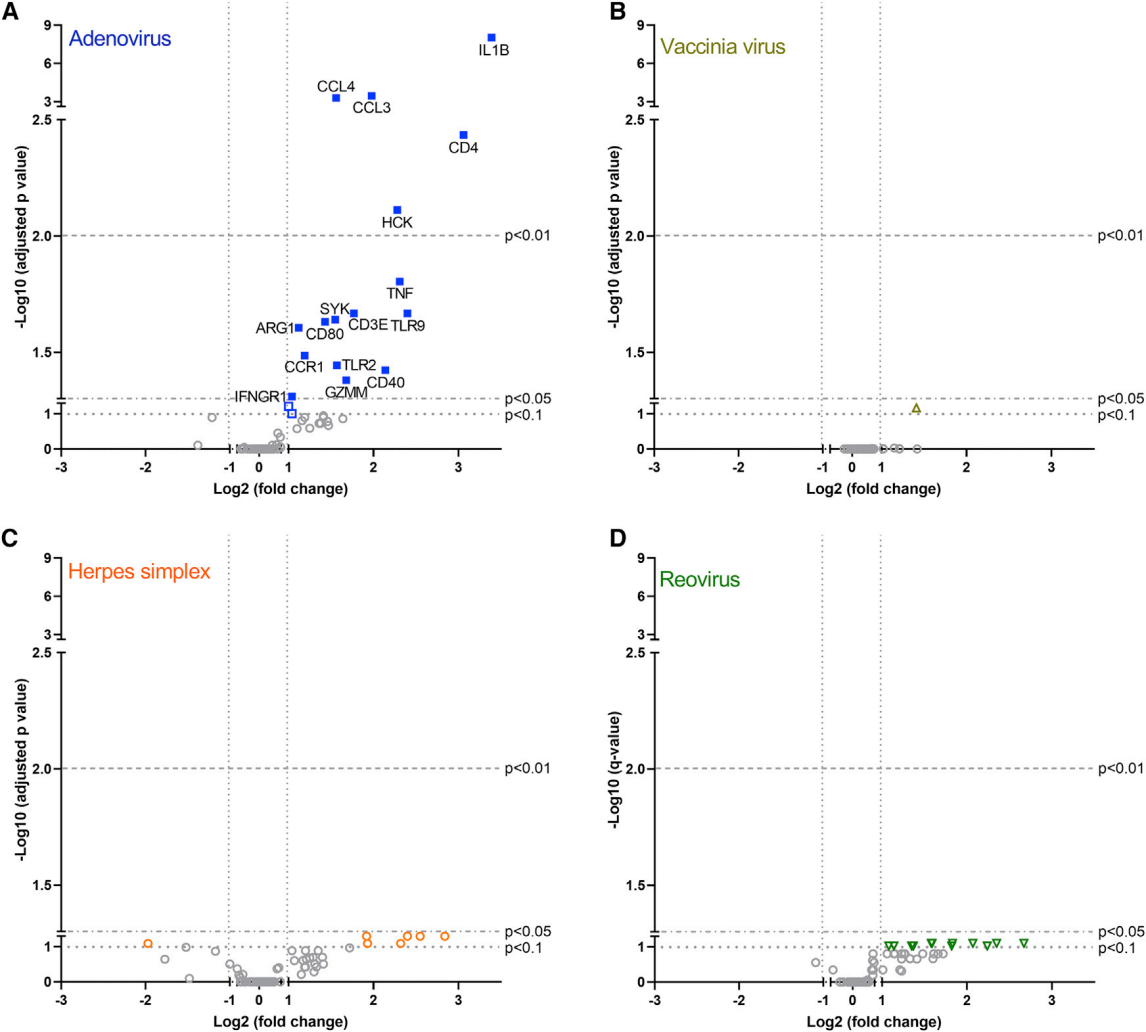
Impact of Different Oncolytic Viruses in the Tumor Microenvironment Comparisons were made between RNA expression profiles from the different virally treated groups versus PBS-treated group. (A–D) Viral treatment used in the group compared to PBS-treated animals: (A) adenovirus, (B) vaccinia virus, (C) herpes simplex virus, and (D) reovirus. The plots indicate the names of those genes for which there is a statistically significant difference (adjusted p value < 0.05) and an expression change of at least double or half compared to reference group (−1 > log_2_ fold change > 1).

Animals receiving a T cell transfer might obtain an improved outcome from the therapy if the tumor microenvironment is altered to favor the development and exertion of immune responses against malignant cells. To study this hypothesis, tumors collected at day 6 (Figure 3A) were analyzed for gene-expression levels. RNA was extracted and studied by a multiplexed immune panel, specifically designed for Syrian hamsters (Table S1). From all of the designed probes, only those that passed quality control were included in the comparison of expression profiles (Figure 4). Adjusted p values and fold changes are described in Table S2.

Gene-expression profiles after treatment with virotherapy and T cell therapy showed that the only candidate able to induce significant changes in the studied genes was adenovirus. In adenovirus-treated tumors, genes related to the following functions were upregulated: production of proinflammatory cytokines (*IL1B*, *TNF*, *GZMM*), proinflammatory cytokine receptors (*IFNGR1*), innate immune system (*TLR9*, *TLR2*), myeloid stimulatory markers (*CD80*, *CD40*), chemokines (*CCL3*, *CCL4*), chemokine receptors (*CCR1*), adaptive immunity cell markers (*CD4*, *CD3E*), and immune signal mediators (*HCK*, *SYK*). This points to a broad immune activity being stimulated at the tumor niche, covering a wide range of immune mechanisms. In addition, an upregulation of *ARG1* (a gene coding for immunosuppressive arginase 1) was also witnessed, possibly indicating a counter-reaction to immunostimulation.

The other three viruses studied in this experiment did not produce any significant upregulation in the genes included in the panel, although subsignificant gene-expression variations were seen in reovirus and herpes simplex virus groups (Table S2). For the tumors treated with vaccinia virus, there were barely any gene-expression alterations even at a subsignificant level.

### An Engineered Adenovirus Armed with TNF-α and IL-2 Increases Efficacy

**Figure 5:**
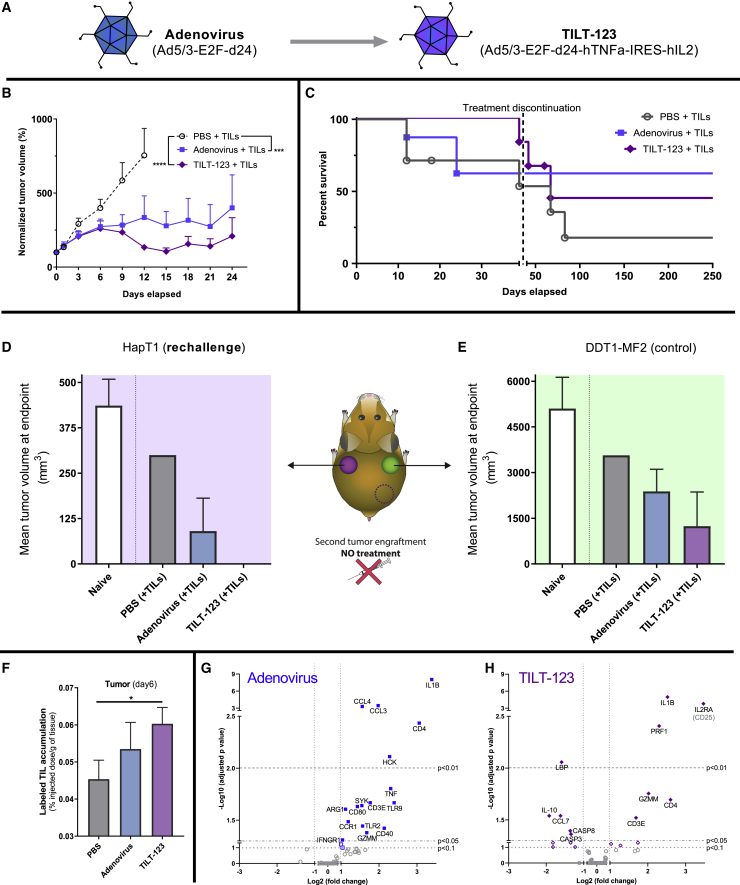
Antitumor and Immunological Effects of Arming an Adenovirus with Immunostimulatory Cytokines (A) Summary of unarmed and armed virus constructs. (B) Grouped-normalized tumor volume values for the different groups (n = 6–9 per group) after receiving TIL therapy (intraperitoneally) and virotherapy or PBS (intratumorally). Treatment schedule is presented in Figure 1A. (C) Overall survival data. Gray dashed line marks discontinuation of the treatments. (D) Mean tumor volume at day 19 after HapT1 rechallenge in complete responders from HapT1 tumors. (E) Mean tumor volume at day 19 after DDT1-MF2 challenge in complete responders from HapT1 tumors (naive, n = 3; PBS + TILs, n = 1; adenovirus + TILs, n = 5; TILT-123 + TILs, n = 2). (F) 13 Syrian hamsters carrying subcutaneous HapT1 tumors were randomized into groups and treated with ^111^indium-labeled TILs intraperitoneally and with PBS (n = 4), unarmed adenovirus (n = 5), or armed adenovirus (n = 4). At day 6 of the experiment, tumors were harvested for *ex vivo*^111^indium measurement by a gamma counter (∗p < 0.05). Comparisons were made between the RNA expression profiles from each of the two adenovirus-treated groups versus the PBS-treated group. Animals carrying these tumors were treated as described in Figure 3A. Viral treatment used in the group compared to PBS-treated animals: (G) adenovirus and (H) TILT-123. The plots indicate the names of those genes for which there is a statistically significant difference (adjusted p value < 0.05) and an expression change over double or below half to those in the reference group (−1 > log_2_ fold change > 1). All error bars are SEM.

As adenovirus appeared the best candidate to enhance ACT among the studied viruses, we studied differences between an unarmed virus and a cytokine-armed virus (Figure 5A). TILT-123 is armed with tumor necrosis factor (TNF)-α and interleukin (IL)-2 (Ad5/3-E2F-d24-hTNFa-IRES-hIL2),^48^^,^^49^ selected for their ability to boost antitumor activity in the T cell compartment.^48^

When comparing antitumor efficacy in the experimental set-up, described in Figure 1A, TILT-123 performed better in terms of tumor growth control than the unarmed adenovirus (adenovirus versus PBS, p = 3.41 × 10^−4^; TILT-123 versus PBS, p = 1.15 × 10^−9^) (Figure 5B). Survival data (Figure 5C) show how the armed version of the virus provided better survival for the first 40 days, but after treatment discontinuation, tumor progression led to the death of many animals (nonsignificant difference).

Complete responders were rechallenged, as described before (Figure 2A). Although the number of animals was limited (n = 2), TILT-123-treated animals showed total rejection, specifically for HapT1 tumors (Figures 5D and 5E). These data seem to indicate improved antitumor efficacy and immunological memory with cytokine-armed oncolytic adenovirus over the unarmed adenovirus.

When studying the impact of the arming device on the trafficking of the adoptive T cell graft, there was statistically significant increased trafficking when compared to mock (p < 0.046), in contrast with the unarmed adenovirus (Figure 5F).

We also studied how the arming device affects the tumor’s gene-expression profile. When the expression patterns of armed and unarmed adenovirus tumors were directly compared, the only genes significantly downregulated in the TILT-123-treated group were *CCL4* (adjusted p value = 0.045; fold change = 0.436) and *TNF* (adjusted p value = 0.045; fold change = 0.201) (Figure S3). When comparing the changes induced by each of those viruses as opposed to the PBS injection, some of the changes were seen with both viruses (*IL1B*, *CD4*, *CD3E*, *GZMM*), whereas other genes that were unaffected by the unarmed virus were upregulated (*IL2RA*, *PRF1*) or downregulated (*LBP*, *IL-10*, *CCL7*, *CASP3*, *CASP8*) upon TILT-123 treatment (Figures 5G and 5H). Additionally, several of the upregulated genes with the unarmed virus were not induced with the armed virus (*CCL4*, *CCL3*, *HCK*, *IFNGR1*, *CD40*, *CD80*, *TLR2*, *TLR9*, *CCR1*, *SYK*, *ARG1*, *TNF*).

## Discussion

The implementation of immunotherapies, such as checkpoint inhibitors and various immune-cell, therapy-based platforms, has mediated a therapeutic revolution in oncology. At the same time, there is clear room for improvement, as the patients responding to immunological treatments are still the minority,^26^^,^^50^ with some exceptions (e.g., CAR T therapies in some hematological malignancies or anti-programmed cell death-1 [PD1] in selected indications). Oncolytic viruses offer a rational alternative to solve the limitations of those immunotherapies, but the lack of head-to-head comparisons between different virus platforms has slowed optimization of the approach. In addition, given major differences in the biology of popular oncolytic viruses, there might be different optimal uses for each. For that reason, this study focuses on a direct comparison of some of the most relevant candidates in the context of TIL therapy.

When comparing different therapeutic options, the challenge is to select the optimal dosing for each treatment. One possible option is to use the same dose for all of the viruses. For that approach, the first limitation is that different viruses are usually titered by different parameters: adenoviruses in vps, herpes simplex and vaccinia virus in PFUs, and reovirus in TCID_50_. Although these differences could, in theory, be sorted out by assembling a set of viruses and measuring their properties, the conversion between units would not be applicable to multiple virus batches, thus invalidating references to previous work. Another critical limitation when using the same dose for all of the viruses is the fact that different viruses have different effects in the host, which is translated into different therapeutic windows. Those virus-caused effects occur at various levels, including at the immune compartment. For this reason, we narrowed down the comparison of the viruses only in the context of T cell therapies and not going into a deep characterization of the viruses as monotherapies.

The approach almost universally used in oncology is that drugs are used at their maximum tolerated dose. Different oncolytic viruses are used in the clinics at different therapeutic ranges, so taking this information into account also helps to obtain more realistic results with regard to safety versus efficacy. In that sense, and together with the results showing that the 10-fold increase in dose did not produce a stronger antitumor effect, we considered that the doses extrapolated weight per weight from the maximum-tolerated human dose were appropriate.

Adenovirus emerged as the strongest candidate for enabling adoptive T cell therapy. Tumors responding to treatment within the first 21 days (5/8) did not progress afterward, and eventually showed complete responses. The large amount of complete responders (62.5% of all of the animals receiving TILs and adenovirus compared to 17.9% of those receiving TILs alone) highlights the potential of adenoviruses to enable T cell antitumor functions. Other viruses did not provide significant improvements in tumor growth control or survival. However, some parameters, such as median survival, were increased when compared with the PBS control (108 days for reovirus versus 67 days for PBS, not statistically significant).

In vaccinia virus, herpes simplex virus, and reovirus-treated groups, responses to the treatment were observed in individual animals, but unlike in the adenovirus group, some of the tumors that shrank to a barely palpable volume eventually progressed and reached the maximum tumor dimensions allowed. In this particular experiment, the herpes simplex group was the only one that did not show any complete responses. Further engineering of the HSV could help to have a higher local antigen presentation (infected cell protein (ICP) 47 deletion) and higher local immunostimulatory effect (gamma34.5 deletion) in the tumor milieu.^51^

As oncolytic viruses have a dual mechanism of action (direct oncolysis and immune-mediated antitumor response), we wanted to assess what is the immune outcome of each oncolytic virus. For that purpose, animals that developed complete responses against pancreatic carcinoma tumors were challenged again with the same cell line and left untreated. The only animals that completely rejected the tumors were those belonging to the adenovirus treatment group. Eighty percent of the animals once cured by oncolytic adenovirus and TIL therapy showed fully protective antitumor-specific memory, whereas none of the other animals (previously cured with TILs alone or in combination with vaccinia virus or reovirus) managed to reject the graft. Other studies focused on the systemic effect after the use of oncolytic adenoviruses and T cell therapies, showing that metastatic processes can be avoided^52^ and that uninjected, established tumors benefit from the treatment.^53^ These results enabled us to hypothesize that oncolytic viruses can deliver complete responses by either direct oncolysis or by the development of immune-related effects. Whereas the development of adaptive responses against the tumor is enhanced by oncolysis,^39^^,^^40^ meaning that the variables are not really independent, it is, at least theoretically, possible that some oncolytic viruses deliver antitumor responses mainly by direct oncolysis. This would imply that cell lysis by those viruses would not enhance effective generation of antitumor-specific immune responses. The dual mechanism of action does not need to be seen from a binary perspective but to be understood in a sense that each virus generates both mechanisms to different extents. Of note, our data suggest that there are important differences between viral strains in this regard.

After the observation of tumor-specific immune responses, it was interesting for us to understand the contribution of the T cell graft and the overall immune microenvironment to those responses. T cell graft biodistribution data did not support a hypothesis where the outcome of the therapies could be solely explained by trafficking. A major limitation of this technique could arise from the intratumoral proliferation of infiltrated radiolabeled T cells. For example, the percentage of the initially injected dose measured by SPECT/CT would be the same regardless of whether T cells newly arrived to the tumor would remain nonproliferative or expand several rounds. Because the radioactivity inside cells cannot increase, a nonproliferative cell would give the same absolute radioactive signal as the sum of, e.g., 8 cells resulting from 3 rounds of mitosis of the initial cell, whereas the actual amount of T cells is 8 times higher (Figure S4). Still, by taking this possibility into account, the values and significances from the early time points in the biodistribution assay do not endorse trafficking as the main mechanistic explanation as to why the tumors treated with the oncolytic adenovirus respond better to adoptive T cell therapy than other groups. It is also relevant to emphasize that the trafficking of endogenous T cells to the tumor was not measured, and that can play an important role in terms of antitumor efficacy. Moreover, we did not measure possible *de novo* adaptive responses, also known as epitope spreading, which are known to occur following adenovirus injection into tumors.^48^

The investigation of expression levels of immunologically relevant genes from tumors complements the T cell graft-trafficking data. Previous studies highlighted the ability of adenoviruses to engage successfully with the immune system, in particular, with the T cell compartment.^54^, ^55^, ^56^ Following those ideas, the results presented in this study show how adenovirus—uniquely among the studied viruses—triggers a wide immunostimulatory response. Genes upregulated by treatment related to innate immunity elements (*TLR9*, *TLR2*), myeloid cell markers of activation (*CD80*, *CD40*), lymphocyte markers (*CD3E*, *CD4*), and their cytotoxic effector components (*GZMM*), diverse extracellular and intracellular messengers of immune stimulation (*IL1B*, *TNF*, *IFNGR1*, *HCK*, *SYK*), and proinflammatory chemokines for the attraction of lymphocytes (*CCL3*) and macrophages (*CCL4*). This molecular network points to a circumstance where it is very likely that innate immunity promotes an eventual appearance of adaptive immunity against the tumor. In this sense, the use of oncolytic viruses seems to have a broad effect in the tumor microenvironment that includes not only interactions with the T cell compartment of the immune system but also a diverse number of cell types. The dissection of the effect of the virus on the different cell types in the tumor would be a way to study further the mechanism on how oncolytic viruses can enhance T cell therapies.

The study of whole tumor-expression patterns was relevant to understand the overall situation at the tumor, although it would be valuable to have a deeper understanding of the contribution of those changes, specifically by different cell types (i.e., tumor cells, dendritic cells, T cells, etc.).

In addition, we saw upregulation of *ARG1*, which codes for arginase, an enzyme that attenuates T cell activity.^57^ The induction of *ARG1* can be a homeostatic response to balance the upregulation of the other 15 immunostimulatory genes.^58^^,^^59^ One of the limitations of this experiment is that it has been set to a specific time point (day 6 after treatment started), which might give an incomplete view of the immune effects triggered earlier or later. However, day 6 should be an adequate time point to study the coexistence of both innate and adaptive immune processes, as adaptive responses usually take 4–7 days to be developed. These results go in line with previous work by others,^60^, ^61^, ^62^ where they show the importance of having a favorable tumor microenvironment that supports antitumor immune responses.

Modern oncolytic virotherapy is gaining relevance as a therapeutic approach, due to the possibilities it offers in terms of immune modulation. Several oncolytic viruses that are currently under clinical and preclinical development are engineered to express immune mediators, such as cytokines, immune ligands, or antagonists. TILT-123 is a 5/3 chimeric adenovirus, designed specifically to enable the T cell compartment of the immune system.^49^ Because the adenovirus performed better than the other viruses as a T cell enabler, it was particularly attractive to compare the treatment outcome with an adenovirus engineered to enhance specifically such a cell population. The arming device had a positive impact on reducing tumor size and increasing antitumor-specific memory and T cell trafficking toward the tumor. Interestingly, there was less significant upregulation of immunostimulatory genes compared with the unarmed adenovirus, as previously described by Havunen et al.^53^ In addition, treatment induced some immunosuppressive genes, such as *IL-10*, which again might be a homeostatic response.

Reovirus and herpes simplex virus showed the fastest direct oncolysis of tumor cells but delivered subsignificant tumor microenvironment modifications, which if reinforced or optimized, could end up improving the overall outcome of the therapy. Vaccinia seemed to have a less visible impact at the immunological level, maybe because even if tumor selective, it is a virus naturally armed with a considerable armamentarium for immune evasion.^63^^,^^64^ Another possibility for the absence of changes in the immune microenvironment after some of the viruses is that the intrinsic tumor-immune suppressivity could dampen the signals produced by those viruses, and only the most potent ones would be able to overcome that suppressivity threshold. Nevertheless, vaccinia viruses, together with herpes simplex viruses, have the largest genome space for the insertion of transgenes. Thus, a comparison of viruses armed with immunostimulatory molecules could yield different results. Another layer of complexity is added when taking into account that different viruses have faster or slower oncolytic cycles, which affects both immunogenicity and direct tumor cell killing.

An important issue regarding the use of oncolytic viruses is the length of treatment. In the case of the comparison between armed and unarmed adenovirus, it seems that as long as the therapy was ongoing, a higher proportion of tumors were under control with TILT-123 treatment. When the therapy was discontinued, survival decreased to a similar level as with the unarmed group. One interpretation of this phenomenon could be linked to the fact that the virus is designed to express the transgenes only while the virus is actively replicating.^49^ If tumor fate is still undecided in terms of antitumor versus protumor forces having ongoing cytokine production (transgene expression is bound to replication), then this might play an important role that is ablated as soon as the virus is cleared. This would not be the case for other sources of antitumor effects, such as the immune responses generated after the oncolytic cycle. It can be argued that long-term dosing could be required in clinical trials using viruses with an arming device. This approach was employed in a phase 3 trial with talimogene laherparepvec, when treatment continued for up to 18 months if efficacy was seen.^65^ Another rationale supporting the multiple administration of the virotherapy relates to the fact that as antiviral immunity builds up, it reduces the persistence of the virus, but it can be helpful to boost immune activity inside the tumor.^66^ In summary, armed and unarmed adenoviruses appear appealing for enabling T cell therapy.

This study did not intend to compare the direct oncolytic activity of the different viruses but instead, how they could be used in the context of T cell therapy. For the use of oncolytic viruses to enable other types of therapies, a different experimental design would be required. In this study’s specific context, oncolytic adenoviruses seemed the strongest candidate among those tested. Higher antitumor effects correlated with changes in the tumor microenvironment. Interestingly, modification of the microenvironment appeared more important than effects on T cell trafficking. The results also make evident the opportunities arising from oncolytic viruses interacting with the immune system to favor antitumor responses. The tailoring of a T cell-specific arming device in an adenovirus (such as TNF-α and IL-2 used here) can help improve the immunostimulatory capacities of these viruses even further. TILT-123 is now being studied in melanoma patients receiving a TIL therapy (ClinicalTrials.gov: NCT04217473).

## Materials and Methods

### Oncolytic Viruses

Oncolytic viruses from four different virus families were included in this study: Ad5/3-E2F-d24 (*Adenoviridae*), VVtd-tomato (JX-929 strain) (*Poxviridae*),^67^ HSV-1 (17+)Lox-PmCMVGFP (*Herpesviridae*; a kind gift from Beate Sodeik, Hannover Medical School, Germany),^68^^,^^69^ and Pelareorep (Reolysin) (*Reoviridae*; a kind gift from Oncolytics). An engineered version of the adenovirus candidate (Ad5/3-E2F-d24-hTNFa-IRES-hIL2, also known as TILT-123) was also used in the experimental phase. Intratumoral administration of the viruses in 50 μL of PBS (or PBS alone in the control groups) was performed by direct injection with 30G insulin needles, according to the schedule established for each experiment.

### Animal and Tumor Model

Male Syrian hamsters (French colony) were used as an animal model for the *in vivo* experimentation. They were obtained as 4–6 weeks old from Charles River Laboratories (Wilmington, MA, USA). The Syrian hamster model was selected to study *in vivo* efficacy, as it is one of the few models permissive for the replication of all of the viruses used in the study.^42^, ^43^, ^44^, ^45^ The tumor model selected is the syngeneic HapT1 pancreatic carcinoma, as it allows growing TILs *ex vivo* to be used as a model for ACT. A syngeneic pancreatic carcinoma cell line (Hap-T1) was used to studying the antitumor efficacy of the treatments in the study. For that purpose, 2 × 10^6^ cells were subcutaneously delivered into the lower lateral flank(s) of the hamsters. 5–6 days later, when tumors were palpable and measurable, they started receiving treatments. Animals, whose tumors surpassed the maximum-tolerated tumor dimensions, were euthanized and marked as dead. Animals developing ulcers were marked as censored in survival studies and euthanized. When studying antitumor memory, those animals showing complete responses from the originally engrafted tumor (lower-right flank) were rechallenged with 2 × 10^6^ HapT1 cells in the upper-left flank and 2.5 × 10^5^ DDT1-MF2 (syngeneic leiomyosarcoma cell line) in the upper-right flank to evaluate the specificity of the antitumor memory. For the tumor-volume records, a digital caliper was used to measure tumor dimensions and transformed into volume by using a standardized formula (0.5 × longest diameter × shortest diameter^2^).

### Adoptive Cell Therapy Treatments

TILs were generated out of HapT1 tumors and used as an adoptive cell therapy graft, as described before.^46^ Briefly, HapT1 tumors are grown until they reach a diameter close to 20 mm. At that point, tumors are harvested and cultured in immunostimulatory conditions (IL-2 and concanavalin A) to expand the TILs present in the tumor. After expansion, T cells are collected, and 4 × 10^7^ cells are intraperitoneally administered to the animals on what is considered day 0 of the experiments.

### Biodistribution Analyses

TILs were labeled with ^111^In-oxine, as described earlier, and administered intraperitoneally into Syrian hamsters.^53^ The injected dose was 4.82 ± 0.72 MBq. The hamsters were bearing two HapT1 tumors (n = 3–4/group). Animals were imaged with NanoScan SPECT/CT (Mediso, Budapest, Hungary) at 24, 72, and 144 h after the administration of the radiolabeled cells. For *in vivo* measuring of the TIL trafficking and accumulation, tumors were delineated by using the coregistered CT images. The results were calculated as a percentage of activity in the tumor from the injected dose. Corresponding values from the biodistribution data at day 6 were divided by the tissue mass (grams). On day 6, tumors were harvested after *in vivo* imaging and the radioactivity was measured *ex vivo* by a gamma counter (Wizard 3; Perkin Elmer, Waltham, MA, USA) for validation. Similarly, CT-defined tumor volumes were correlated with *ex vivo* mass to validate the approach. Other tissues, such as blood, kidney, spleen, and muscle, were also harvested for *ex vivo* measurement of radioactivity.

### Gene-Expression Analyses

Tumors harvested during *in vivo* experimentation were stabilized in RNAlater (R0901; Sigma-Aldrich, St. Louis, MO, USA) and stored at −20°C. RNA was purified from those tumors following the RNeasy (74104; QIAGEN, Hilden, Germany) kit manufacturer’s guide. Total RNA concentration was measured in all of the samples with a BioPhotometer (Eppendorf, Westbury, NY, USA) to ensure the presence of sufficient RNA concentration. For quantitative assessment of the expression of 96 genes, we designed a custom nCounter panel (NanoString Technologies, Seattle, WA, USA) that was run for the samples, as indicated by manufacturer. Data analysis was performed by Nanostring’s data analysis service (single-blind analysis), where normalization of gene expression, based on housekeeping genes, was performed. Quality-control checkups were performed for all of the samples and all of the target genes included in the panel. Differential expression in genes between oncolytic virus-treated tumors and PBS-treated tumors was represented in volcano plots based on the significance and fold change for each gene.

### Oncolytic Activity Measurements

HapT1 cells were cultured *in vitro* up to 14 days in the presence of the above-mentioned viruses at different concentrations. The concentrations used are relative to the viral doses described before. Cell viability was assessed with the CellTiter 96 AQueous One Solution Cell Proliferation Assay (G3582; Promega, Madison, WI, USA), following the manufacturer’s indications. The viability of mock-treated cells was set to 100%.

### Statistics

SPSS Statistics 25 software (IBM, Armonk, NY, USA) was used to perform a mixed-model analysis for tumor-growth evolution based on the tumor volumes (logarithmic transformation of the volumes normalized on day 0 volumes), as described before.^70^ GraphPad Prism 8 (GraphPad Software, San Diego, CA, USA) was used for log rank Mantel-Cox test on Kaplan-Meier survival curves, Pearson’s r, linear regression, as well as the graphic representation of the data. p values under 0.05 were considered statistically significant

### Ethical Statement

Based on the recommendations included in the Act on the Protection of Animals Used for Scientific or Educational Purposes (497/2013) and the government decree on the Protection of Animals Used for Scientific or Educational Purposes (564/2013), as well as the European Directive 2010/63/EU, experimental protocols and procedures were established and then approved by the ethical committee of the Animal Experimental Board (ELLA) of the Regional State Administrative Agency of Southern Finland.

## Author Contributions

Conception and design, V.C.-C., M.S., and A.H.; Development of Methodology, V.C.-C., J.M.S., D.C.A.Q., J.H.A.C., T.K., D.L., M.V., and M.A.; Acquisition of Data, V.C.-C., E.K., D.C.A.Q., J.H.A.C., J.M.S., R.H., T.K., D.L., and M.A.; Analysis and Interpretation of Data, V.C.-C., D.C.A.Q., J.M.S., R.H., M.S., T.K., A.J.A., and A.H.; Writing – Review & Editing, V.C.-C., D.C.A.Q., R.H., J.M.S., E.K., J.H.A.K., M.S., S.Z., C.H., T.K., D.L., M.V., A.G.-H., A.J.A., S.S., M.A., V.H., A.K., and A.H.; Administrative, Technical, or Material Support, A.G.-H., A.J.A., M.A., V.H., A.K., and A.H.

## Conflicts of Interest

A.H. is shareholder in Targovax ASA (Oslo, Norway) and in TILT Biotherapeutics (Helsinki, Finland). A.H., M.S., R.H., S.S., J.M.S., and V.C.-C. are employees of TILT Biotherapeutics. Other coauthors declare no competing interests.

## Supplemental Information

Document S1. Tables S1 and S2 and Figures S1–S4

Document S2. Article plus Supplemental Information
