# Supplementary material for: Comparison of Clinically Relevant Oncolytic Virus Platforms for Enhancing T Cell Therapy of Solid Tumors
Source: Mol Ther Oncolytics. 2020 Mar 19;17:47–60. doi: 10.1016/j.omto.2020.03.003 (PMC7163046; doi:10.1016/j.omto.2020.03.003)
Supplement: Document S1. Tables S1 and S2 and Figures S1–S4 [file mmc1.pdf]

OMTO, Volume *17*

Supplemental Information

Comparison of Clinically Relevant Oncolytic Virus Platforms for Enhancing T Cell Therapy of Solid Tumors

Victor Cervera-Carrascon, Dafne C.A. Quixabeira, Riikka Havunen, Joao M. Santos, Emma Kutvonen, James H.A. Clubb, Mikko Siurala, Camilla Heiniö, Sadia Zafar, Teija Koivula, Dave Lumen, Marjo Vaha, Arturo Garcia-Horsman, Anu J. Airaksinen, Suvi Sorsa, Marjukka Anttila, Veijo Hukkanen, Anna Kanerva, and Akseli Hemminki

Supplemental information

Gene	Accession code	Position	Tm CP (ºC)	Source	QC check
ARG1	NM_001281645.1	594-693	84	Reference	Pass
ARG2	XM_005072738.2	948-1047	83	Predicted	Fail
ATM	XM_013112839.1	3324-3423	78	Predicted	Pass
BAX	XM_005084711.2	400-499	84	Predicted	Pass
BID	XM_013127166.1	127-226	79	Predicted	Pass
BTLA	XM_013116855.1	1216-1315	81	Predicted	Fail
CASP3	NM_001281582.1	229-328	83	Reference	Pass
CASP8	XM_005070606.2	1272-1371	83	Predicted	Pass
CCL1	XM_005076964.1	155-254	84	Predicted	Fail
CCL17	XM_005078618.2	200-299	85	Predicted	Fail
CCL2	XM_005076967.2	355-454	81	Predicted	Pass
CCL22	NM_001281664.1	142-241	83	Reference	Fail
CCL3	NM_001281338.1	118-217	80	Reference	Pass
CCL4	XM_005076934.2	120-219	81	Predicted	Pass
CCL5	XM_005076936.2	153-252	80	Predicted	Pass
CCL7	XM_005076968.2	129-228	85	Predicted	Pass
CCL8	XM_005077080.2	406-505	83	Predicted	Pass
CCR1	XM_005082282.2	492-591	82	Predicted	Pass
CCR3	XM_013122202.1	540-639	78	Predicted	Pass
CCR4	NM_001281562.1	428-527	81	Reference	Fail
CCR5	XM_005082278.2	320-419	85	Predicted	Pass
CD2	XM_005076665.2	315-414	82	Predicted	Fail
CD27	XM_005084050.2	1091-1190	84	Predicted	Pass
CD274	XM_005063709.2	370-469	84	Predicted	Pass
CD28	XM_013113684.1	364-463	84	Predicted	Fail
CD3E	XM_013112878.1	376-475	83	Predicted	Pass
CD4	XM_005065933.2	53-152	83	Predicted	Pass
CD40	XM_005084980.2	601-700	83	Predicted	Pass
CD40LG	XM_005084522.2	595-694	83	Predicted	Fail
CD80	NM_001281419.1	644-743	81	Reference	Pass
CD86	NM_001310555.1	655-754	79	Reference	Pass
CD8A	XM_005076083.1	303-402	81	Predicted	Pass
CX3CL1	XM_005078617.2	162-261	85	Predicted	Pass
CXCL10	NM_001281344.1	289-388	80	Reference	Pass
CXCL11	XM_005068143.1	91-190	84	Predicted	Pass

Gene	Accession code	Position	Tm CP (ºC)	Source	QC check
FOXP3	XM_005085088.2	1149-1248	83	Predicted	Fail
FUT7	XM_013123264.1	642-741	84	Predicted	Pass
GAPDH	XM_013124485.1	171-270	86	Predicted	Fail
GZMH	XM_013124876.1	65-164	83	Predicted	Fail
GZMK	XM_005065528.1	231-330	78	Predicted	Pass
GZMM	XM_013122967.1	990-1089	86	Predicted	Pass
HAVCR2	XM_005071890.2	294-393	83	Predicted	Pass
HCK	XM_005086067.2	1299-1398	84	Predicted	Pass
HMGB1	XM_013122822.1	228-327	84	Predicted	Pass
IDO1	XM_005066560.2	551-650	83	Predicted	Pass
IFNAR1	XM_013116152.1	1206-1305	80	Predicted	Pass
IFNG	NM_001281631.1	417-516	83	Reference	Pass
IFNGR1	XM_005065730.2	657-756	81	Predicted	Pass
IL10	XM_005079860.1	188-287	83	Predicted	Pass
IL12A	NM_001281367.1	647-746	83	Reference	Fail
IL12B	NM_001281689.1	349-448	79	Reference	Fail
IL13	XM_005067910.2	208-307	85	Predicted	Fail
IL15	XM_005077725.2	685-784	80	Predicted	Fail
IL1B	XM_005068610.2	488-587	82	Predicted	Pass
IL2	NM_001281629.1	440-539	81	Reference	Fail
IL21	XM_005069697.1	126-225	79	Predicted	Fail
IL25	XM_013124877.1	474-573	86	Predicted	Fail
IL2RA	XM_005071734.1	426-525	83	Predicted	Pass
IL4	XM_005067769.1	144-243	83	Predicted	Fail
IL6	XM_005087110.1	274-373	83	Predicted	Pass
IL9	XM_005078463.1	229-328	82	Predicted	Fail
IRAK1	XM_013125875.1	888-987	80	Predicted	Pass
IRAK4	XM_013111016.1	1463-1562	84	Predicted	Pass
IRF1	NM_001281646.1	1090-1189	83	Reference	Pass
IRF2	XM_013110855.1	439-538	79	Predicted	Pass
IRF3	XM_005084741.2	810-909	80	Predicted	Pass
IRF4	XM_005066422.2	223-322	84	Predicted	Pass
IRF5	XM_013119090.1	1450-1549	84	Predicted	Pass
IRF8	XM_005073104.2	592-691	85	Predicted	Pass
ITGA1	XM_013122573.1	2325-2424	82	Predicted	Pass

Gene	Accession code	Position	Tm CP (ºC)	Source	QC check
JAK1	XM_005086635.2	3531-3630	80	Predicted	Pass
JAK2	XM_005063713.2	2659-2758	81	Predicted	Pass
KLRG1	XM_005066036.2	420-519	82	Predicted	Pass
LBP	XM_005082653.2	915-1014	84	Predicted	Pass
LTA	XM_005086798.1	355-454	83	Predicted	Fail
MYD88	XM_005082348.2	877-976	83	Predicted	Pass
OAS3	XM_013119800.1	1273-1372	83	Predicted	Pass
PECAM1	XM_013113224.1	1241-1340	79	Predicted	Pass
PNMA1	XM_005086397.2	1094-1193	86	Predicted	Fail
PRF1	XM_005070839.2	1470-1569	83	Predicted	Pass
PRKCE	XM_005077299.2	2718-2817	82	Predicted	Fail
SBNO2	XM_005083326.2	1741-1840	86	Predicted	Pass
SYK	XM_013110528.1	1532-1631	80	Predicted	Pass
TGFB1	XM_013125593.1	1035-1134	81	Predicted	Pass
TGFB2	XM_005082438.2	747-846	80	Predicted	Pass
THBD	XM_005072559.2	1393-1492	83	Predicted	Pass
TICAM1	XM_005088421.2	1865-1964	83	Predicted	Pass
TIGIT	XM_005074799.1	83-182	82	Predicted	Pass
TLR2	XM_005080078.2	798-897	83	Predicted	Pass
TLR3	XM_005066656.2	1181-1280	83	Predicted	Pass
TLR9	XM_013116942.1	1405-1504	77	Predicted	Pass
TNF	XM_005086799.2	223-322	82	Predicted	Pass
TNFSF10	XM_005069757.2	476-575	80	Predicted	Pass
TYK2	XM_005078559.2	3590-3689	82	Predicted	Pass
VEGFA	NM_001281843.1	458-557	81	Reference	Pass
VEGFC	XM_005066687.2	1557-1656	83	Predicted	Pass

***Supplementary table 1. List of genes analyzed in the multiplexed RNA profiling of Syrian hamster tumors**. Tm CP; Temperature of melting – Crossing point. QC check; Quality control check.*

Adenovirus			Vaccinia virus			Herpes simplex virus			Reovirus		
**Gene name**	**Adj. P-value**	**Fold change**	**Gene name**	**Adj. P-value**	**Fold change**	**Gene name**	**Adj. P-value**	**Fold change**	**Gene name**	**Adj. P-value**	**Fold change**
IL1B	9.16E-09	10.5									
CCL3	0.00036	3.95									
CCL4	0.000504	2.96									
CD4	0.00368	8.34									
HCK	0.00774	4.84									
TNF	0.0157	4.95									
TLR9	0.0215	5.28									
CD3E	0.0215	3.41									
SYK	0.0229	2.92									
CD80	0.0234	2.7									
ARG1	0.0248	2.17									
CCR1	0.0327	2.28									
TLR2	0.036	2.97									
CD40	0.0378	4.41									
GZMM	0.0417	3.21									
IFNGR1	0.049	2.06									
CD274	0.0613	2.01	CD274	0.0672	2.66	CD4	0.0503	7.14	TLR9	0.0788	6.34
THBD	0.0991	2.06				CD3E	0.0503	3.8	GZMM	0.0788	4.2
						TLR9	0.0503	5.84	CD3E	0.079	3.55
						IL2RA	0.0503	5.27	CCL5	0.079	3
						IL10	0.0807	0.256	CD40	0.079	5.11
						CD40	0.0807	4.98	CD8A	0.079	2.99
						HCK	0.0807	3.81	HAVCR2	0.0937	2.58
									HCK	0.0937	3.53
									CD27	0.0937	2.54
									CCR1	0.0937	2.21
									IFNGR1	0.0937	2.11
									CD4	0.0937	4.71

<-- Significance threshold

Supplementary table 2. Significance and fold change of the gene expression in the comparison of virally treated tumors versus control. *Data shown for the changes in genes with an adjusted p value <0.1.*

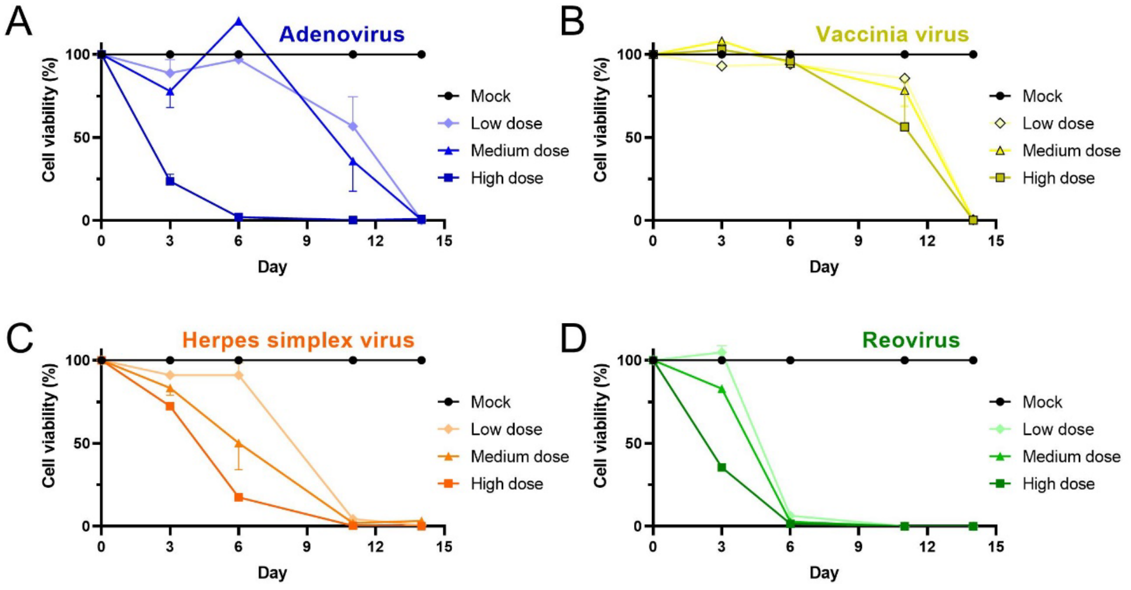

Supplementary figure 1. Oncolytic capability of the viruses. *HapT1 cells were cultured with three concentrations of viruses extrapolated from in previously described treatments (using each virus standard units) and cell viability was measured at days 3, 6, 11 and 14. (A) Adenovirus: 10^2, 10^3 and 10^4 vp/cell. (B) Vaccinia virus: $5*10^{-2}$, $5*10^{-1}$ and 5 pfu/cell. (C) Herpes simplex virus: $7*10^{-3}$, $7*10^{-2}$ and $7*10^{-1}$ pfu/cell. (D) Reovirus: $5*10^{-1}$, 5 and $5*10^1$ $TCID_{50}$/cell. (All error bars showed are SEM).*

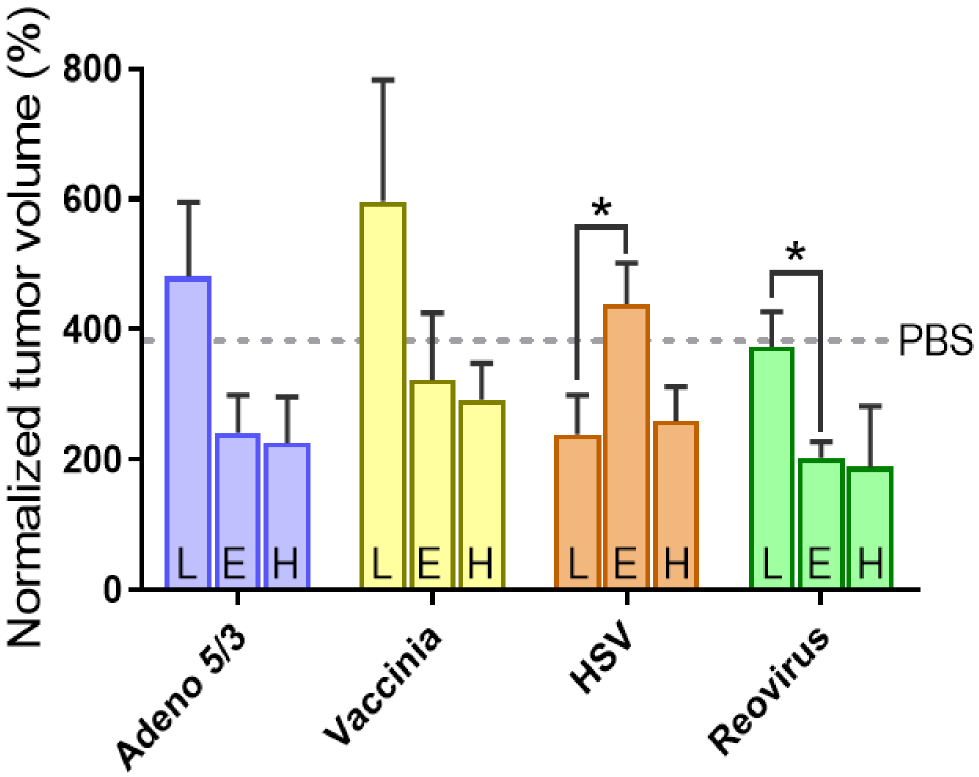

Supplementary figure 2. Assessment of the dose-effect differences for different oncolytic viruses. *39 Syrian Hamsters engrafted with bilateral subcutaneous HapT1 tumors were randomized and assigned to different groups treated intratumorally with PBS or different oncolytic viruses at different doses (L; 0.1x extrapolated dose. E; 1x extrapolated dose. H; 10x extrapolated dose). Each group comprised 6 tumors. Mean normalized tumor volume for each group is shown (with SEM). The grey dashed line represents mean normalized tumor volume for a negative control group.*

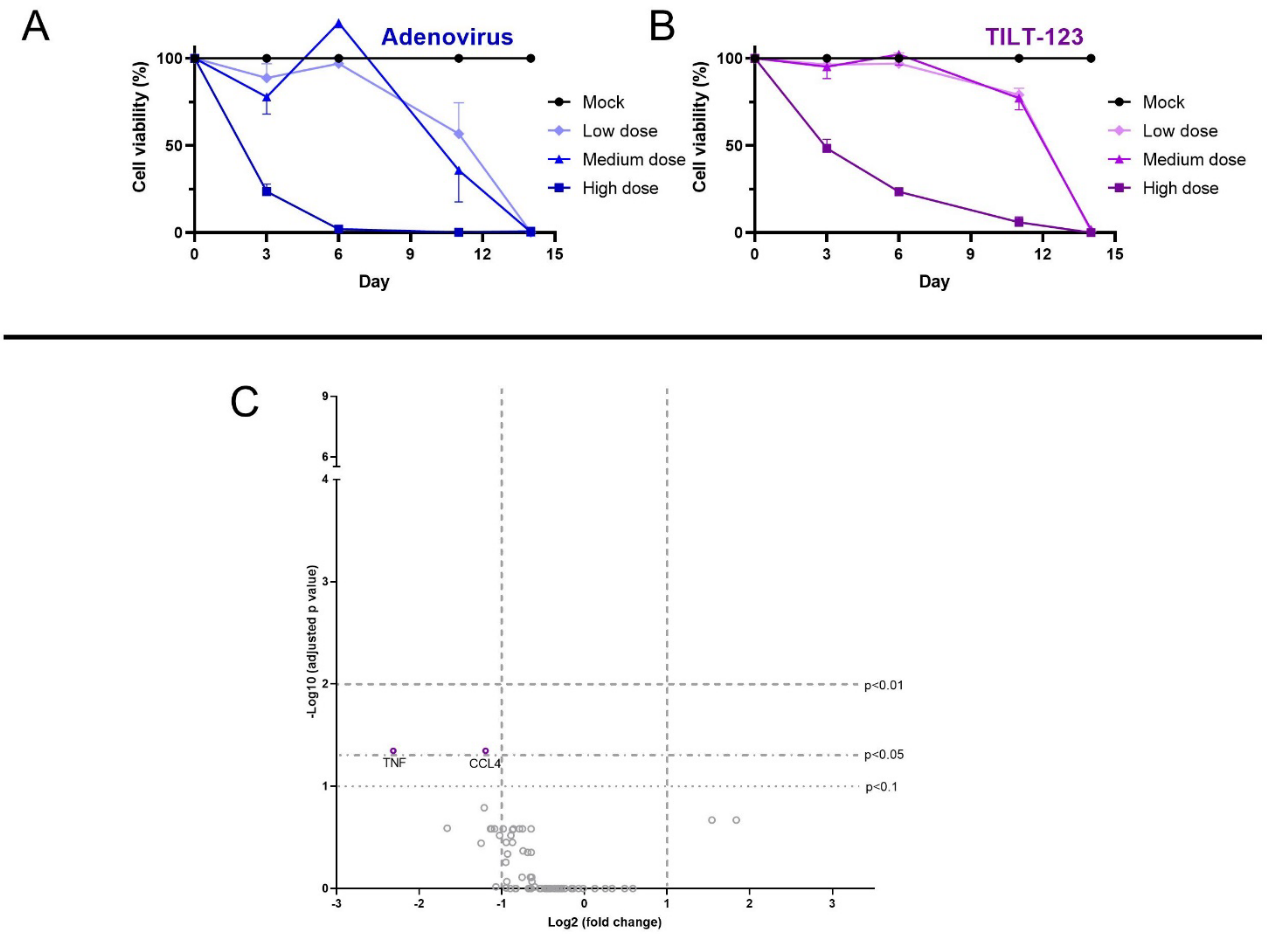

Supplementary figure 3. Impact of the TNFa and IL-2 arming deviceon oncolytic activity and gene expression. *(A-B) HapT1 cells were cultured with three concentrations of both adenoviruses and cell viability was measured at days 3, 6, 11 and 14.* 10^2*,* 10^3 *and* 10^4 *vp/cell of each virus was used. (C) A gene expression comparison for TILT-123 against unarmed adenovirus was made. The plot indicates the name of those genes for which there is a statistically significant difference (adjusted p value <0.05) and an expression change of at least double or half compared to reference group (-1>*Log_2 *fold change>1). (All error bars showed are SEM).*

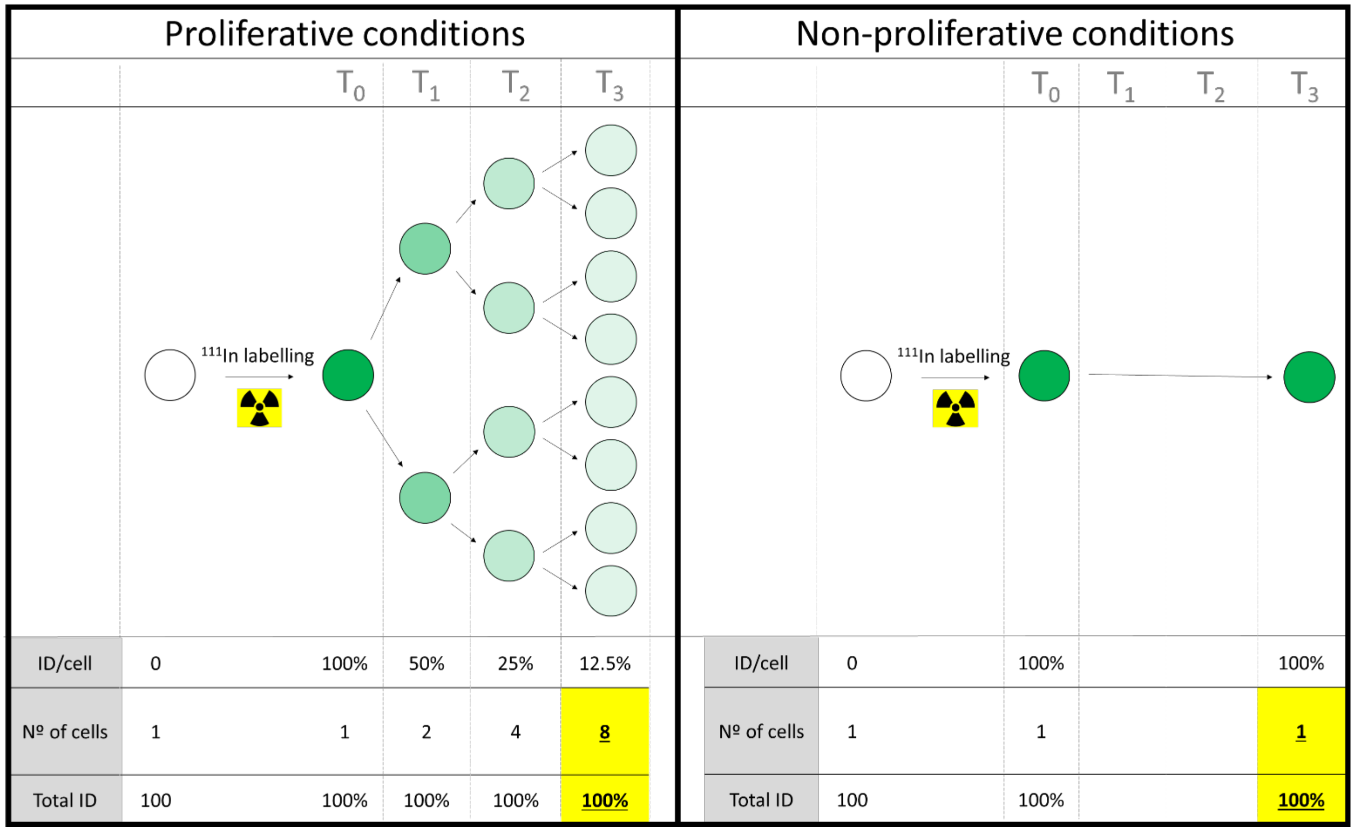

Supplementary figure 4. Limitations of radiolabel-based biodistribution after cell proliferation. *When labeled cells start proliferating, the effector abilities of all the cells combined increases, the total radioactivity signal remains the same and the signal per cell decreases proportionally.* ^{111}*In; Radioactive Indium . ID; injected dose.*
